# Exploiting individual differences to assess the role of place and phase locking cues in auditory frequency discrimination at 2 kHz

**DOI:** 10.1038/s41598-023-40571-1

**Published:** 2023-08-23

**Authors:** Vinay, Brian C. J. Moore

**Affiliations:** 1 Audiology Group, Department of Neuromedicine and Movement Science, Faculty of Medicine and Health Sciences, Norwegian University of Science and Technology (NTNU), Tungasletta 2, 7491 Trondheim, Norway; 2 Cambridge Hearing Group, Department of Psychology, University of Cambridge, Cambridge, UK

**Keywords:** Auditory system, Cochlea, Hair cell, Inner ear

## Abstract

The relative role of place and temporal mechanisms in auditory frequency discrimination was assessed for a centre frequency of 2 kHz. Four measures of frequency discrimination were obtained for 63 normal-hearing participants: detection of frequency modulation using modulation rates of 2 Hz (FM2) and 20 Hz (FM20); detection of a change in frequency across successive pure tones (difference limen for frequency, DLF); and detection of changes in the temporal fine structure of bandpass filtered complex tones centred at 2 kHz (TFS). Previous work has suggested that: FM2 depends on the use of both temporal and place cues; FM20 depends primarily on the use of place cues because the temporal mechanism cannot track rapid changes in frequency; DLF depends primarily on temporal cues; TFS depends exclusively on temporal cues. This led to the following predicted patterns of the correlations of scores across participants: DLF and TFS should be highly correlated; FM2 should be correlated with DLF and TFS; FM20 should not be correlated with DLF or TFS. The results were broadly consistent with these predictions and with the idea that frequency discrimination at 2 kHz depends partly or primarily on temporal cues except for frequency modulation detection at a high rate.

## Introduction

The auditory detection of changes in frequency may depend on two mechanisms. The first, called the “place” mechanism, is based on the frequency analysis that occurs in the cochlea^1,2^. The place of maximum response along the cochlear partition varies with input frequency and changes in frequency may be coded by changes in the place of maximum response^1^ or by changes on the low-frequency (apical) side of the response pattern, also called the excitation pattern^3,4^. In principle, the place mechanism can operate over the whole audible frequency range. The second mechanism, called the “temporal” mechanism, depends on the fact that, at least for low-frequency inputs, the action potentials (spikes) evoked in auditory neurons are synchronised to a specific phase of the input waveform, a property called phase locking^5^. As a result, the time intervals between successive spikes are approximately integer multiples of the period of the input. Changes in frequency may be detected via changes in the pattern of inter-spike intervals^6–8^. It is known that phase locking in the auditory nerve of animals becomes less precise at high frequencies^5,9,10^. Such a loss of precision probably also occurs for humans, but the upper limit of phase locking in humans is still a subject of considerable debate. Some researchers have argued that the temporal mechanism plays a role in frequency discrimination for frequencies up to 8–10 kHz^7,8,11,12^, while others have argued for a much lower limit, perhaps 1.5 kHz^13,14^.

Frequency discrimination can be measured in several ways. One method is to measure the modulation depth required to distinguish a frequency-modulated carrier from an unmodulated carrier^3,15–17^. The threshold determined in this way is called the frequency modulation detection limen (FMDL). It has been proposed that the detection of frequency modulation (FM) depends partly on the use of temporal (phase-locking) information when the carrier frequency is below about 5 kHz and the FM rate is low (below about 5 Hz), while for FM rates above about 10 Hz only a place mechanism is used^18–23^. This proposal is based on the idea that the mechanism that “decodes” the temporal information is “sluggish” and cannot track rapid changes in frequency^16,19^.

The relative importance of place and temporal information for low FM rates may depend on the overall level of the stimuli. Ernst and Moore^24^ measured FMDLs for carrier frequencies (*f*_c_) of 1000, 4000, and 6000 Hz, using modulation frequencies (*f*_m_) of 2 and 10 Hz and levels of 20 and 60 dB SL, both with and without random amplitude modulation (AM), applied in all intervals of a forced-choice trial. The AM was intended to disrupt place cues for detection of the FM. At 60 dB SL, the deleterious effect of the AM was smaller for *f*_m_ = 2 than for *f*_m_ = 10 Hz for *f*_c_ = 1000 and 4000 Hz, but not for *f*_c_ = 6000 Hz. This is consistent with the idea that, for *f*_c_ below about 5000 Hz and *f*_m_ = 2 Hz, frequency modulation can be detected via changes in phase locking over time and this information is less affected than place information by the addition of random AM. However, at 20 dB SL, the deleterious effect of the added AM for *f*_c_ = 1000 and 4000 Hz was similar for the two values of *f*_m_, while for *f*_c_ = 6000 Hz, the deleterious effect of the AM was greater for *f*_m_ = 10 than for *f*_m_ = 2 Hz. Ernst and Moore^24^ suggested that, at low SLs, the auditory filters become relatively sharp, so that excitation-pattern cues influence FMDLs to a greater extent, even for low *f*_c_ and low *f*_m_. However, the hypothesis that FM detection depends partly on temporal information for low FM rates and mainly on place information for high rates is not universally accepted^25–27^.

Another measure of frequency discrimination is the smallest detectable difference in frequency between successive steady tones^28^. This measure is called the difference limen for frequency (DLF). In one version of this measure, two successive tones are presented with slightly different frequencies and the participant is asked to indicate whether the first or the second is higher in pitch. However, untrained participants often find it hard to name the direction of a pitch change, and extensive practice may be required to achieve stable performance^29–31^. A task that is easier and requires less practice involves the use of four successive tones in each of two observation intervals. In one randomly selected interval all four tones have the same frequency. In the other interval, the tones alternate in frequency between two values, A and B, giving the pattern ABAB. The task is to identify the interval in which the tones changed in pitch^12,32^. Moore and Ernst^12^ used this task over a wide range of centre frequencies. They found that DLFs, expressed as a proportion of centre frequency, worsened with increasing frequency from 2 to 8 kHz and then became roughly constant with further increases in frequency. They suggested that the worsening in DLFs from 2 to 8 kHz reflected a progressive reduction in the precision of temporal information (phase locking) and that the “break point” around 8 kHz reflected a transition from a temporal code to a place code. However, this interpretation is controversial^14^.

Finally, frequency discrimination can be measured using a task that is specifically designed to limit the use of place cues, promoting performance based on phase locking. The task, called the TFS1 task, involves the detection of changes in the temporal fine structure (TFS) of bandpass filtered complex tones^33–35^. The participant is required to discriminate a harmonic complex tone (H), with fundamental frequency F0, from a similar tone in which all components are shifted up in frequency by ΔF (where ΔF ≤ 0.5F0), to create an inharmonic tone (I). People with normal hearing perceive the H and I tones as having different pitches if ΔF is sufficiently large^36–38^. The H and I tones have the same envelope repetition rate (equal to the F0 of the H tones), but they differ in their TFS. The phases of the components are chosen randomly for each H and I tone, with the result that the envelope shape fluctuates randomly from one tone to the next, so that the envelope shape does not provide a cue for discriminating the H and I tones. The H and I tones are made up of many components and are passed through a fixed bandpass filter centred on the higher components, to make place cues minimal. The filter has a central flat region and skirts that decrease in level at a rate of 30 dB/octave. The use of a relatively shallow slope ensures minimal place cues as components move in and out of the passband^38^. Hence, it is thought that performance of the TFS1 task depends primarily on the use of temporal cues rather than place cues^39,40^. The task is similar to that used by Ernst and Moore^12^ to measure DLFs. One randomly selected interval contains the sequence HHHH and the other contains the sequence HIHI, and the participant is asked to identify the interval in which the tones changed in pitch.

The present study was designed to test the following hypotheses, all for a centre frequency of 2 kHz, for which there is debate about the relative importance of place and temporal cues^14^:

1. FMDLs depend partly on a temporal mechanism for an FM rate of 2 Hz, while FMDLs depend primarily on a place mechanism for an FM rate of 20 Hz.
2. DLFs depend primarily on a temporal mechanism.
3. Thresholds measured using the TFS1 task, denoted TFS, depend primarily on a temporal mechanism.

To test these hypotheses, four measures of frequency discrimination were obtained for 63 normal-hearing participants: detection of FM using a 2-Hz modulation rate (FM2); detection of FM using a 20-Hz rate (FM20); DLF; and TFS. There are natural variations across normal-hearing participants in sharpness of tuning^41,42^, which partly determines the efficacy of place cues, and according to the hypotheses these variations should affect FM2 and FM20 values but have little effect on DLF and TFS values. There are also individual variations in the precision of phase locking^43,44^, and according to the hypotheses these should affect the values of DLF and TFS, and to a lesser extent FM2, but should have little effect on FM20 values. The three hypotheses lead to the following predictions based on the variations in performance across participants:

P1. FM2 and FM20 values should be moderately correlated, because place information may be partly used for both FM rates and because the task is very similar for the two measures.

P2. FM2 values should be moderately correlated with DLF and TFS values, because FM2 values depend partly on the use of temporal cues and DLF and TFS values depend strongly on the use of temporal cues.

P3. FM20 values should not be correlated with DLF or TFS values, because FM20 values depend mainly on place cues while DLF and TFS values depend strongly on temporal cues.

P4. DLF and TFS values should be highly correlated because both depend strongly on the use of temporal cues and because the task is very similar for the two measures.

Predictions P1 to P4 imply that some correlations will be bigger than others. The predictions about the relative sizes of the correlations are presented in the Results section, together with the outcomes.

## Methods

### Participants

All participants were students or researchers at the Norwegian University of Science and Technology in Trondheim, Norway. The participants were recruited via flyers and via the university media channel. All participants had audiometric thresholds better than 20 dB HL (measured using an Otometrics Aurical Otosuite and Telephonics TDH-39P headphones) for all octave-spaced frequencies from 250 to 8000 Hz. Their ages ranged from 20 to 33 years. None of the participants had non-auditory neural conditions and none had any history of ear discharge or pain in their ears. None of the participants reported having neurological problems. Middle-ear function was checked using a Grason-Stadler Tympstar Pro tympanometer; all participants had normal middle-ear function. Participants were paid for participating by being given gift vouchers. Data were obtained for 63 participants. This number is sufficient such that any correlation between scores for two tests greater than 0.21 would be significant at *p* < 0.05, using a directional (one-tailed) test.

The study and methods followed the tenets of the Declaration of Helsinki, and informed consent was obtained from participants after the nature and possible consequences of participation were explained. Approval for the experiments was given by the Regional Committee for Medical and Health Research Ethics (REK) in Norway.

### Apparatus and test procedures

Stimuli were generated using the “PSYCHOACOUSTICS” software^45^ using the built-in 24 bit soundcard of a Microsoft Surface Pro laptop computer. Stimuli were delivered via Sennheiser HDA200 headphones, which have a smooth frequency response in the frequency region around 2 kHz. Testing was carried out separately for each ear of each participant in a sound-attenuating room.

All thresholds were measured with a two-alternative forced-choice procedure using a two-down one-up adaptive procedure to estimate the 71% correct point on the psychometric function. The two observation intervals were marked on the laptop screen by successively lighting up two spatially separated boxes. After the participant had responded, feedback was provided by flashing the correct box, with green for a correct response and red for an incorrect response.

The following measures were obtained.

1. Absolute thresholds for 2-kHz pure-tone signals. The signal duration was 1000 ms including 10-ms raised-cosine ramps. The two observation intervals were separated by 500 ms. The step size was 4 dB until four turnpoints had occurred and 2 dB thereafter. A run continued until eight turnpoints (changes from decreasing to increasing level and vice versa) had been obtained using the 2-dB step size. The threshold was taken as the arithmetic mean of the levels at the last six turnpoints. The threshold was measured twice for each ear, and the two estimates were averaged. For the measures of frequency discrimination, the level of the test stimuli (excluding any background noise) was set to be 30 dB SL, i.e., 30 dB above the absolute threshold.
2. FMDLs using modulation frequencies of 2 and 20 Hz and a carrier frequency of 2 kHz. The signal duration was 1000 ms including 20-ms raised-cosine ramps and the two intervals in a trial were separated by 300 ms. The carrier was unmodulated in one randomly chosen interval and sinusoidally frequency modulated in the other. The starting frequency deviation was 80 Hz, which was chosen to be well above the likely threshold value. The frequency deviation was changed by a factor of 1.95 until two turnpoints had occurred, then by a factor of 1.56 until two more turnpoints had occurred and then by a factor of 1.25. A run continued until eight turnpoints had been obtained using the smallest step size. The threshold was taken as the geometric mean of the frequency deviations at the last eight turnpoints. Two estimates of threshold were obtained for each ear, and the final threshold was taken as the geometric mean of the two. Note that thresholds are expressed as the deviation from the carrier frequency; the overall frequency excursion of the modulated carrier was twice the specified deviation. For example, if the threshold was 10 Hz, the instantaneous frequency of the modulated carrier varied from 1990 to 2010 Hz.
3. DLFs were measured using the task outlined in the introduction. In one interval of a trial (selected randomly), there were four successive 200-ms bursts (including 20-ms raised-cosine ramps) of tone A, with a fixed frequency, f. The bursts were separated by 100 ms. In the other interval, tones A and B alternated, with the same 100-ms inter-burst interval, giving the pattern ABAB. The two intervals were separated by 300 ms. Tone B had a frequency that was higher than that of tone A by Δf Hz. The task of the subject was to choose the interval in which the sound changed across the four tone bursts within an interval. The test tones were presented in a background of threshold equalizing noise (TEN)^46^, which was intended to reduce variations in loudness with changes in frequency; the loudness of the test tones was determined mainly by the signal-to-TEN ratio^47^. The TEN started 300 ms before the first tone burst and ended 300 ms after the last tone burst. The TEN level is specified as the level in a 1-ERB_N_ wide band centred at 1000 Hz, where ERB_N_ stands for the average value of equivalent rectangular bandwidth of the auditory filter at moderate sound levels for listeners with normal hearing^48^. The level of the TEN was set 15 dB below the level of the test tones. The starting value of Δf was 80 Hz, which was chosen to be well above the likely threshold value. The value of Δf was changed by a factor of 1.95 until one turnpoint had occurred, then by a factor of 1.56 until one more turnpoint had occurred and then by a factor of 1.25. A run continued until eight turnpoints had been obtained. The threshold was taken as the geometric mean of the Δf values at the last six turnpoints. Two estimates of threshold were obtained for each ear, and the final threshold was taken as the geometric mean of the two.
4. The stimuli for the TFS1 task were bandpass filtered complex tones. The bandpass filter had a flat top and slopes of 30 dB/oct. The passband width is specified as the width of the flat portion (not the 3-dB down points) and was equal to 222.22 Hz. The tones were either harmonic (H) with a fundamental frequency F0 of 222.22 Hz, or all components were shifted up in frequency by ΔF, giving an inharmonic tone I. The lower edge of the passband was set to 2000 Hz, so the 9th harmonic was the lowest component of the H tone that fell within the passband (9 × 222.22 = 2000). To prevent the detection of combination tones, and to limit the audibility of components falling on the skirts of the passband, the tones were presented in a background of TEN with the same characteristics as for the measurement of DLFs. The level of the TEN was set 15 dB below the overall level of the complex tone, which corresponds to about 10.5 dB below the level of each component within the passband. It was hypothesized that the audible components in the stimulus would not be resolved in the auditory system^49^ and that the task would be performed primarily using temporal cues^39,40^. In one interval of a trial (selected randomly), there were four successive 200-ms bursts (including 20-ms raised-cosine ramps) of tone H. The bursts were separated by 100 ms. In the other interval, tones H and I alternated, with the same 100-ms inter-burst interval, giving the pattern HIHI. The two intervals were separated by 300 ms. The task of the subject was to choose the interval in which the sound changed across the four tone bursts within an interval. The phases of the components were chosen randomly for every tone burst, so the envelope of each tone burst was different and performance of the task could not be based on envelope cues. The starting value of ΔF was 111.11 Hz. This value of ΔF leads to the greatest possible difference between the H and I tones. The value of ΔF was changed by a factor of 1.95 until one turnpoint had occurred, then by a factor of 1.56 until one more turnpoint had occurred and then by a factor of 1.25. If the adaptive procedure called for a value of ΔF greater than 111.11 Hz, the value was kept at 111.11 Hz until two successive correct responses occurred. In practice the limit was reached very rarely. A run continued until eight turnpoints had been obtained. The threshold was taken as the geometric mean of the values of ΔF at the last six turnpoints. Two estimates of threshold were obtained for each ear, and the final threshold was taken as the geometric mean of the two.

For each of the frequency discrimination tasks, the nature of the task was carefully described to each participant, including what to “listen for”. For the FM detection tasks they were asked to pick the interval in which the sounds appeared to “wobble”. For the DLF and TFS tasks, they were told to pick the interval in which the tones changed in pitch. When the initial performance of a participant was erratic or when a participant reported that they were not sure what to listen for, the participant was given practice and further instruction until their performance became stable. The FMDLs were measured first, with FM2 and FM20 tested in random order across participants. Then the TFS and DLF tasks were performed. The ear that was tested first was varied randomly across participants.

## Results

The thresholds for each measure were generally similar for the two ears of a given participant. To reduce the effects of random errors of measurement, the thresholds were averaged across ears (arithmetic means for absolute thresholds, geometric means for the measures of frequency discrimination). Two of the measures of frequency discrimination, FM2 and DLF, were weakly but significantly correlated with the absolute thresholds (FM2, *r* = 0.237, *p* = 0.031; DLF, *r* = 0.211, *p* = 0.048, based on a 1-tailed test, since it was hypothesized that performance would worsen with increasing absolute threshold). However, these correlations are not significant if allowance is made for the calculation of four correlations (based on Bonferroni correction).

In what follows, the patterns of correlation between the different measures of frequency discrimination were examined to assess the extent to which they were consistent with predictions P1–P4. Because there were specific predictions about the direction and magnitude of each correlation, no correction was made for the number of correlations being calculated and the significance of the correlations was assessed using one-tailed tests.

**Figure 1:**
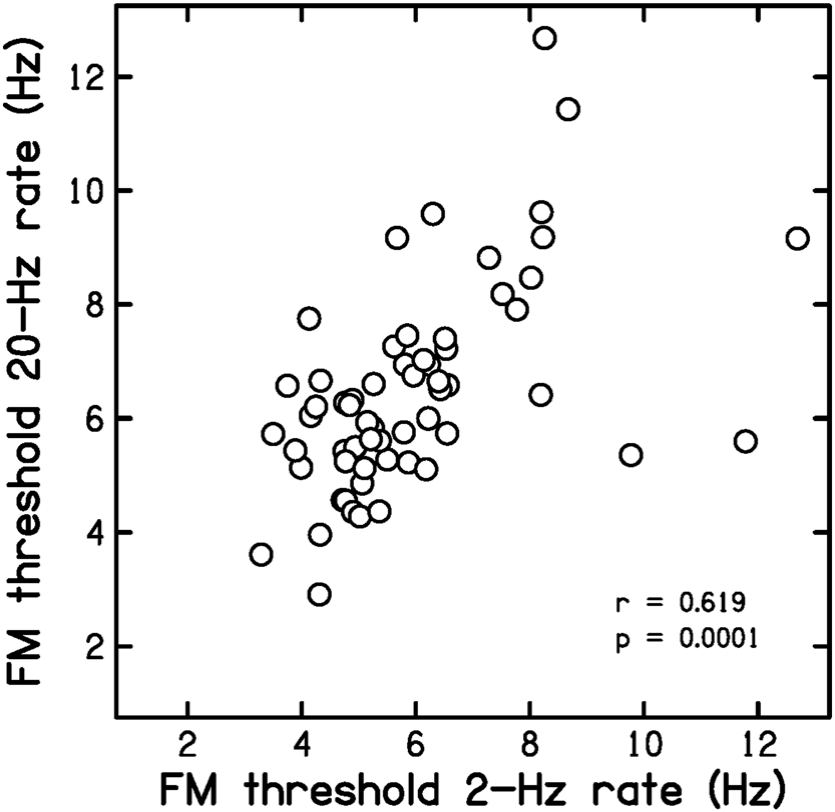
Scatter plot of FM20 values versus FM2 values.

Prediction P1 was that FM2 and FM20 values would be moderately correlated. Figure 1 is a scatter plot of FM20 values versus FM2 values. The correlation between the two was *r* = 0.619 (*p* < 0.0001, 90% confidence interval, CI 0.471, 0.773). Hence P1 was confirmed. The results are consistent with the idea that FM2 and FM20 were partly based on a common mechanism, using place cues.

**Figure 2:**
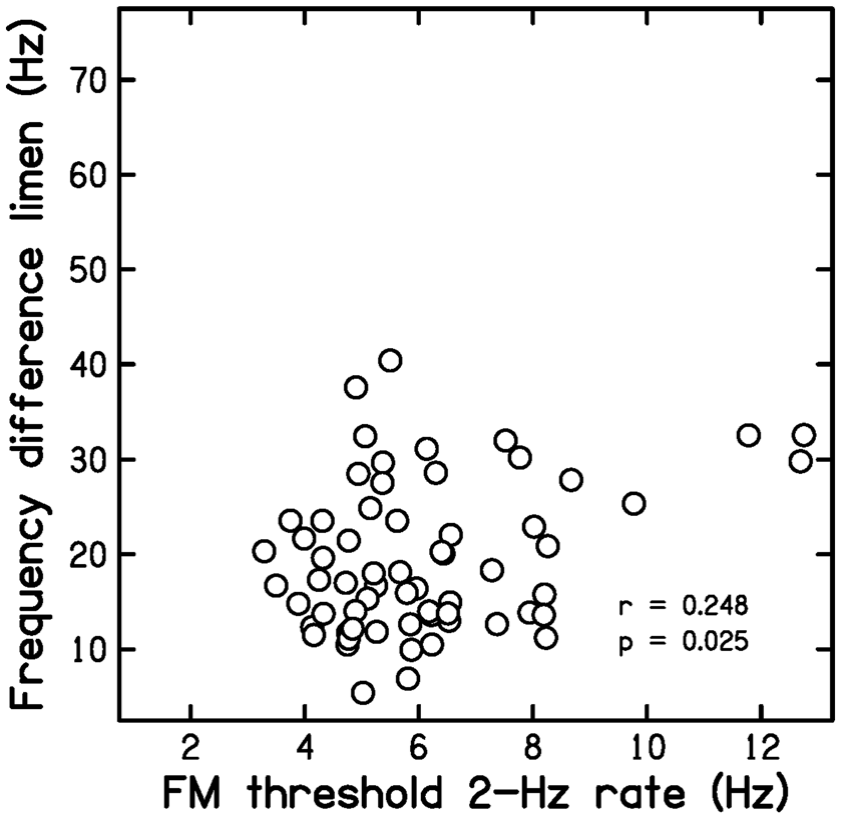
Scatter plot of DLF values versus FM2 values.

Prediction P2 was that FM2 values would be moderately correlated with DLF and TFS values. Figure 2 is a scatter plot of DLF values versus FM2 values. The correlation between the two was *r* = 0.248 (*p* = 0.025, CI 0.041, 0.434).

**Figure 3:**
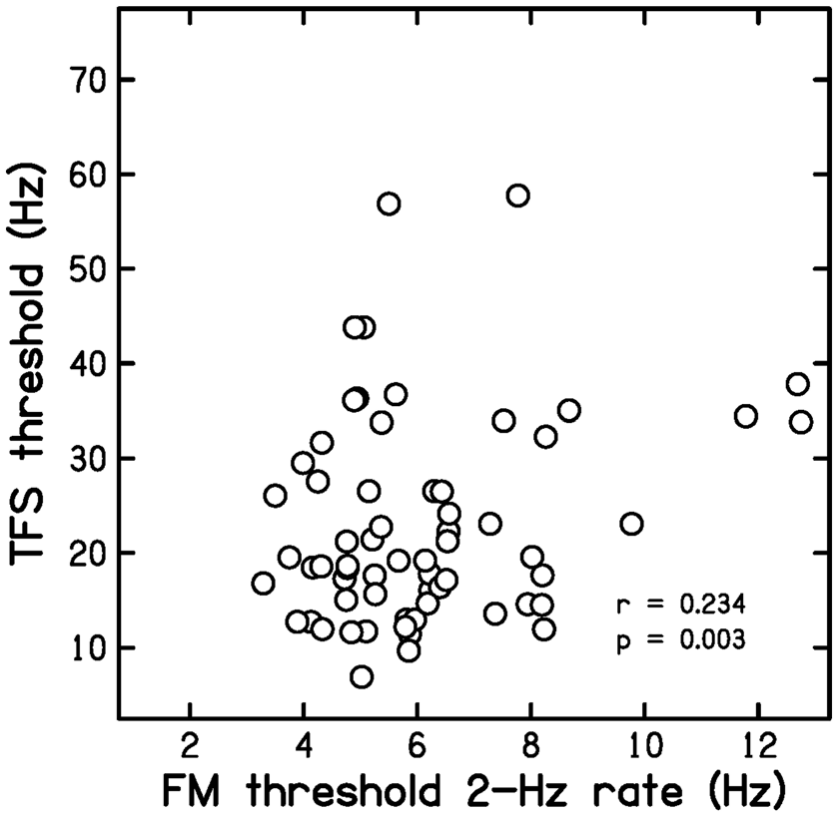
Scatter plot of TFS values versus FM2 values.

Figure 3 is a scatter plot of TFS values versus FM2 values. The correlation between the two was *r* = 0.234 (*p* = 0.032, CI 0.026, 0.423). While the correlations with FM2 values were significant for both the DLF and TFS values, the correlations were small. This weakly supports P2.

**Figure 4:**
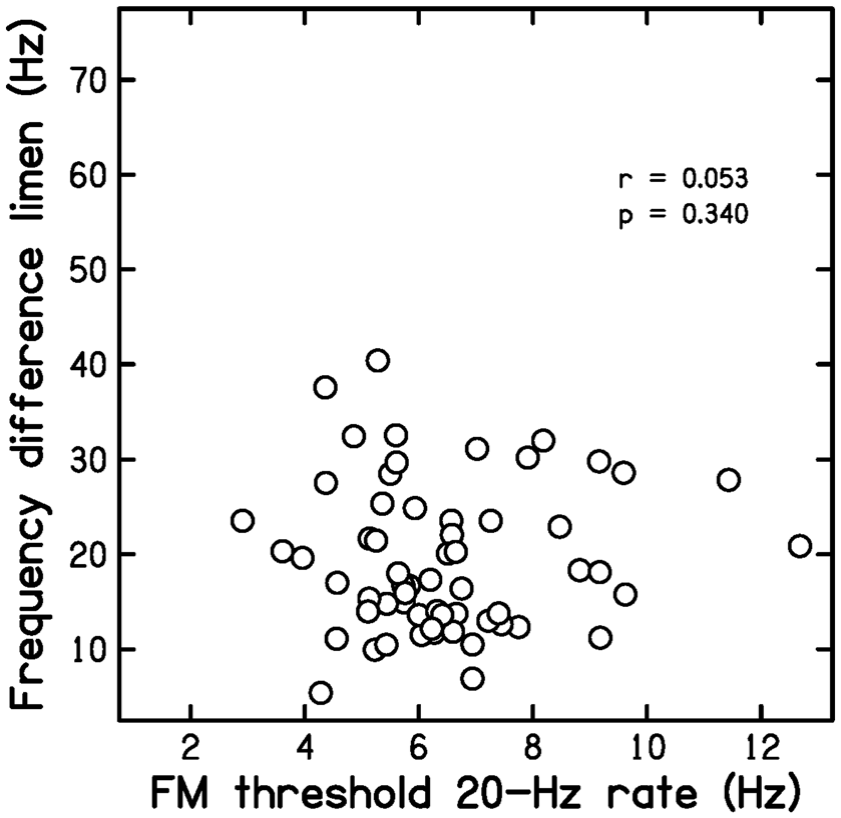
Scatter plot of DLF values versus FM20 values.

**Figure 5:**
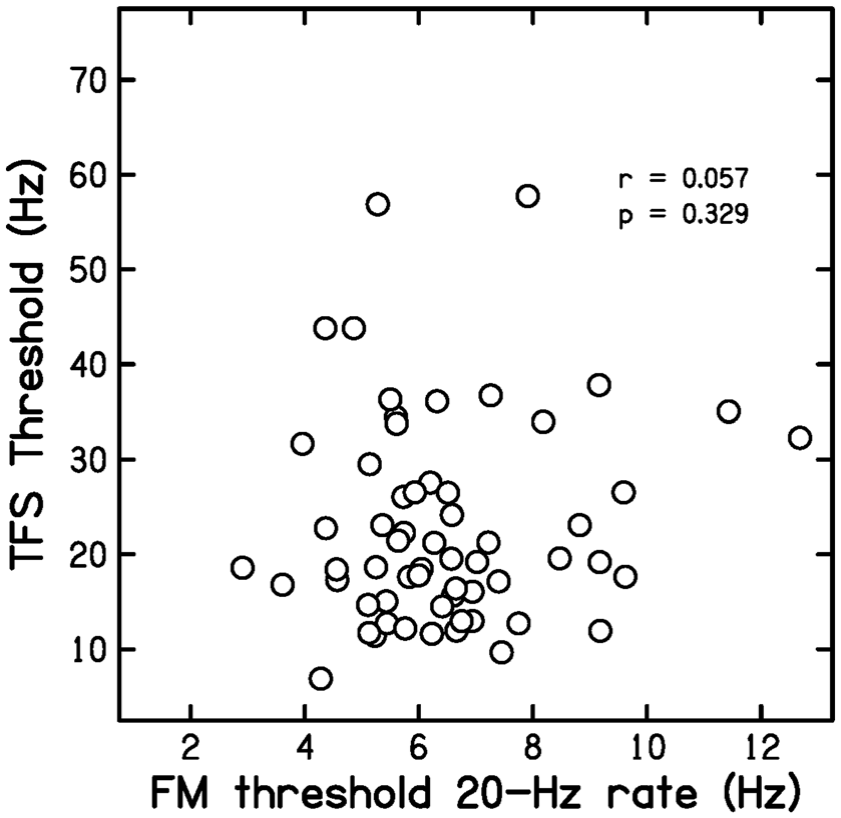
Scatter plot of TFS values versus FM20 values.

Prediction P3 was that FM20 values would not be correlated with DLF or TFS values. Figure 4 is a scatter plot of DLF values versus FM20 values. The correlation between the two was *r* = 0.053 (*p* = 0.340, ns, CI − 0.158, 0.259). Figure 5 is a scatter plot of TFS values versus FM20 values. The correlation between the two was *r* = 0.057 (*p* = 0.329, ns, CI − 0.154, 0.263). These results are consistent with P3.

**Figure 6:**
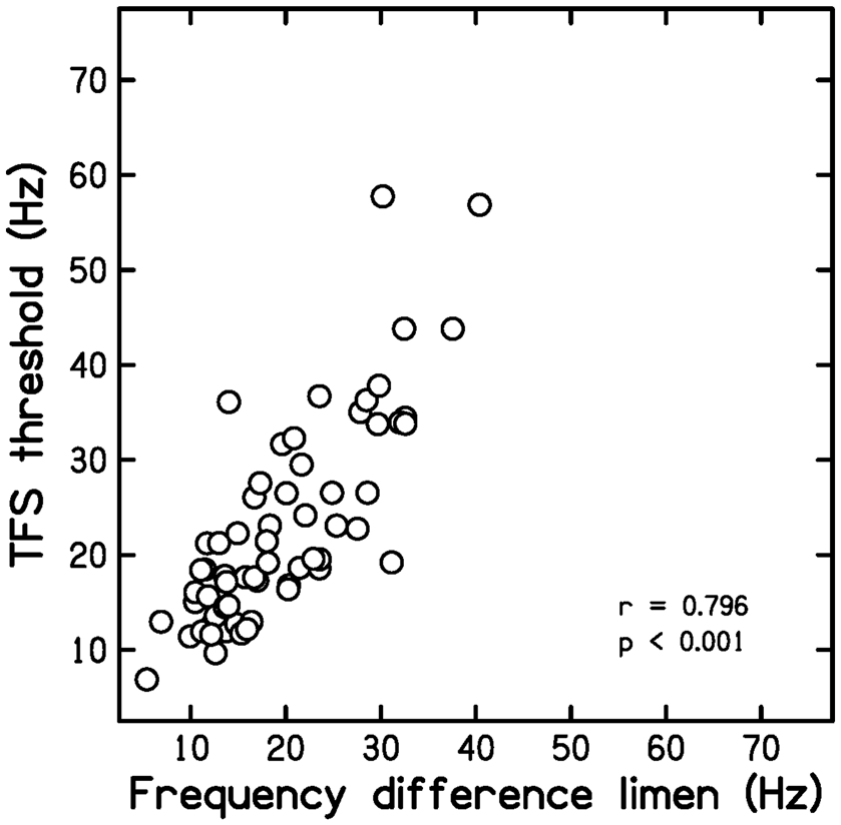
Scatter plot of TFS values versus DLF values.

Prediction P4 was that DLF and TFS values would be highly correlated. Figure 6 is a scatter plot of TFS values versus DLF values. The correlation between the two was *r* = 0.796 (*p* < 0.001, CI 0.704, 0.862). This high correlation is consistent with P4.

As noted in the “Introduction”, predictions P1 to P4 imply that some correlations will be bigger than others. Specifically, the following predictions can be made:

1. *r*(TFS vs DLF) should be greater than *r*(FM2 vs FM20). This is the case because TFS and DLF values are hypothesized to depend primarily on the use of temporal cues, FM2 values to depend partly on temporal cues and partly on place cues, and FM20 values to depend primarily on place cues. The two correlations were 0.796 and 0.619, and based on Fisher’s *r* to *z* transform, these differ significantly (*p* = 0.023).
2. *r*(FM2 vs TFS) should be greater than *r*(FM20 vs TFS). This is the case because TFS values are hypothesized to depend primarily on the use of temporal cues, FM2 values to depend partly on temporal cues and partly on place cues, and FM20 values to depend primarily on place cues. The two correlations were 0.234 and 0.057, which was in the predicted direction, but the difference just failed to reach significance (*p* = 0.055) (significance in this case and the case below was calculated taking into account the fact that the two correlations have a variable in common, in this case TFS^50^).
3. *r*(FM2 vs DLF) should be greater than *r*(FM20 vs DLF). This is the case because DLF values are hypothesized to depend primarily on the use of temporal cues, FM2 values to depend partly on temporal cues and partly on place cues, and FM20 values to depend primarily on place cues. The two correlations were 0.248 and 0.053, and the difference was significant (*p* = 0.039).

Overall, these outcomes are broadly consistent with the predictions.

**Figure 7:**
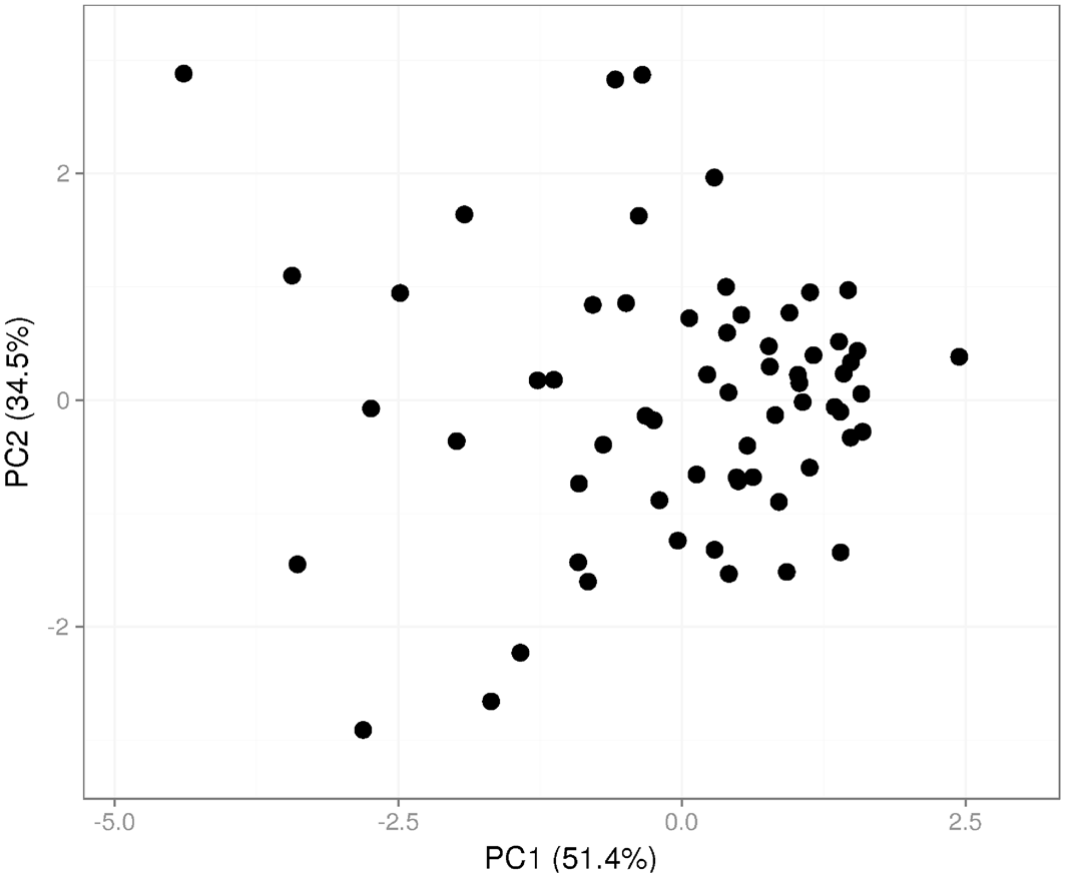
Results of a principal components analysis showing where each participant fell in the PC1–PC2 space.

To gain more insight into the relationship between the scores for the four measures, a principal components analysis was conducted using the “ClustVis software^51^. The first two principal components (PC1 and PC2) accounted for 51.4 and 34.5% of the variance in the data, respectively, the remaining two components accounting for only 14% of the variance. Figure 7 shows where each participant fell in the PC1–PC2 space. There is no obvious clustering.

Table 1 shows the loading of each measure on PC1 and PC2. FM2 loaded moderately on PC1 and PC2. FM20 loaded strongly on PC2 and weakly on PC1. TFS and DLF loaded moderately strongly on PC1 and more weakly negatively on PC2. A reasonable interpretation is that PC1 primarily reflects sensitivity to temporal cues while PC2 primarily reflects sensitivity to place cues. The analysis is consistent with the idea that TFS and DLF depend mainly on the use of temporal cues, FM20 depends mainly on the use of place cues, and FM2 depends on both temporal and place cues.

**Table 1:** Loading of each measure on PC1 and PC2.

	PC1	PC2
FM2	0.49	0.48
FM20	0.35	0.66
TFS	0.55	− 0.43
DLF	0.57	− 0.40

## Discussion

As noted in the introduction, the hypothesis that FM detection depends partly on the use of temporal information for low FM rates but exclusively on place information for high FM rates is the subject of some controversy^25–27,52^. For example, in a study also exploiting individual differences Whiteford and Oxenham^25^ measured FMDLs for 1- and 20-Hz rates and a 500-Hz carrier presented at 70 dB SPL using 100 young normal-hearing participants. They also measured the detection of AM for the same rates, and assessed sensitivity to binaural phase-locking cues using a task involving the discrimination of FM that was in-phase at the two ears from FM that was out of phase at the two ears. This measure is denoted here dichotic FM. The correlation between the 1-Hz FMDLs and the dichotic FM thresholds, was not higher than the correlation between the 20-Hz FMDLs and dichotic FM thresholds, contrary to what would be expected if both the 1-Hz FMDLs and the dichotic FM thresholds depended on phase locking. However, the discrimination of interaural phase may be limited by specific binaural processes and might not provide a good measure of the ability to use monaural phase locking cues^53^. Whiteford and Oxenham^25^ also estimated the sharpness of the upper and lower slopes of the excitation patterns evoked by the stimuli, using forward masking. For most participants, the lower slope was steeper, as expected^54^. The correlation between the steeper of the lower and upper slopes and the FMDLs was moderate and similar for the two FM rates, contrary to what would be expected if FMDLs for the 20-Hz rate depended entirely on the use of place cues, while FMDLs for the 1-Hz rate depended partly on phase-locking cues. However, the slope measure showed very high individual variability, suggesting that it was imprecise. Also, as pointed out by the authors, the slope measure combined with the measure of AM sensitivity did not predict the 1-Hz or 20-Hz FMDLs better than just the measure of AM sensitivity, suggesting no clear relationship between frequency selectivity and either slow- or fast-rate FM detection thresholds. Thus the results of Whiteford and Oxenham do not allow strong conclusions to be drawn about the relative roles of place and temporal cues in FM detection.

The general pattern of the results presented in the present paper was broadly consistent with predictions P1 to P4. Prediction P1, that FM2 and FM20 values would be moderately correlated because both are partly determined by the use of place cues, was supported. Prediction P2 was that FM2 values would be moderately correlated with DLF and TFS values because FM2 values depend partly on the use of temporal cues and DLF and TFS values depend primarily on the use of temporal cues. This prediction was only weakly supported; the correlations were significant but small. Prediction P3, that FM20 values would not be correlated with DLF or TFS values because FM20 values depend mainly on the use of place cues while DLF and TFS values depend mainly on the use of temporal cues, was supported. Both correlations were below 0.06 and both were non-significant. Finally, prediction P4, that DLF and TFS values would be highly correlated because both depend primarily on the use of temporal cues, was strongly supported.

P2, the weakly supported prediction, is based on the assumption that FM2 values depend partly on the use of temporal cues. The weak support for this prediction may be linked to the low SL of the stimuli in the present experiment, which was 30 dB. At low SLs, the auditory filters, which are thought to reflect the sharpness of the filtering in the cochlea^55^, are relatively sharp^54,56^ and this greater sharpness promotes the use of place cues to detect FM, even for low FM rates^24^. If a higher level had been used, the auditory filters would have been less sharp, and there would probably have been more reliance on temporal cues for FM2^24^. If would be informative to repeat the current experiment using a higher SL. This would lead to a clearer test of the hypotheses and predictions presented in this paper.

The interpretation of the results in terms of the use of temporal and place cues is consistent with the results of the principal components analysis. This analysis showed that two components accounted for 86% of the variance in the data. PC1 can be interpreted as primarily related to the use of temporal cues, with moderately high loadings for TFS and DLF, a moderate loading for FM2 and a low loading for FM20. PC2 can be interpreted as primarily related to the use of place cues, with a high loading for FM20, a moderate loading for FM2, and negative loadings for TFS and DLF.

There is, however, an alternative explanation for the pattern of the results. It is possible that the correlations between scores for the different tasks were determined partly or mainly by the similarity of the tasks and/or stimuli. One could interpret PC1 as being related to the rate of frequency changes, higher loadings indicating slower changes. The number of high-low cycles per second was 1.67 Hz for DLF and TFS, 2 Hz for FM2 and 20 Hz for FM20, so the rate of change was monotonically related to the loading on PC1. One could interpret PC2 as being related to the use of gated tone bursts as opposed to continuous frequency changes; the loadings were negative for the two measures that used gated tone bursts (TFS and DLF) and positive for the two measures that used continuous frequency changes (FM2 and FM2). However, some aspects of the results are not entirely consistent with such an interpretation. Firstly, regarding PC1, the number of high-low cycles per second was only slightly lower for DLF and TFS (1.67 Hz) than for FM2 (2 Hz), but the loading on PC1 was higher for TFS (0.55) and DLF (0.57) than for FM2 (0.49). Secondly, regarding PC2, the loading was markedly higher for FM20 (0.66) than for FM2 (0.48), despite the fact that both involved continuous changes. Overall, while we cannot rule out an explanation for the pattern of results in terms of task/stimulus similarity, an interpretation in terms of temporal and place cues seems more reasonable.

The ability to detect FM based on place cues depends on two main factors: the sharpness of tuning in the cochlea, and the ability to detect fluctuations in excitation level at a given place in the cochlea. The sharpness of the excitation pattern evoked by a frequency-modulated carrier does not depend on FM rate provided that the spectral components of the stimulus are not resolved, but the ability to detect AM for a stimulus of fixed duration is markedly better for a 20-Hz rate than for a 2-Hz rate^24,57,58^, probably because there are more modulation cycles in the stimulus at the higher rate. Hence if place cues were exclusively used to detect FM, the FM20 values should be markedly smaller than the FM2 values. In fact, the geometric mean values were 6.5 Hz for FM20 and 5.8 Hz for FM2. This supports the idea that FM2 values were partly determined by the use of temporal information.

The frequency discrimination thresholds showed considerable individual variability, as has been found for a variety of auditory abilities among participants with audiometric thresholds within the “normal” range^59^. The arithmetic means (with standard deviations, SDs, and ranges) of the frequency discrimination measures in the present study were: FM2 6.1 Hz (1.8, 3.3–12.8 Hz), FM20 6.8 Hz (2.3, 2.9–15.8 Hz), DLF 19.5 Hz (8.0, 5.4–37.6 Hz) and TFS 23.3 Hz (11.2, 6.9–57.8). The individual variability probably stems from both peripheral and central factors. However, it seems likely that the measures FM2 and FM20 were influenced at least partly by individual variability in the sharpness of tuning in the cochlea, which can be assessed by measurement of the sharpness of the auditory filters. For young participants with normal audiometric thresholds, the equivalent rectangular bandwidth of the auditory filters for a center frequency of 2 kHz has a mean of about 308 Hz and an SD of 32 Hz^41^. The SD is only about 10% of the mean. It is usually assumed that the steepness of the slope of the excitation pattern is highly correlated with the equivalent rectangular bandwidth of the auditory filter at the corresponding center frequency^3^, so one would expect the SD of the slope also to be about 10% of the mean value. However, the SDs for the FM2 and FM20 measures were 30% and 34% of the mean, respectively, suggesting sources of individual variability other than the sharpness of auditory filtering. One such source of variability is individual differences in the ability to detect fluctuations in excitation level at a given place within the cochlea^58^.

The individual variability for the measures DLF and TFS probably depends at least partly on individual differences in the precision of phase locking or in the ability of central mechanisms to make use of phase-locking information. It is noteworthy that the SDs of the DLF and TFS values were 41% and 48% of the mean, respectively. These percentages are larger than for the FM2 and FM20 values, suggesting that individual variability in the use of temporal cues is greater than individual variability in the use of place cues. The precision of phase locking can be indirectly assessed by recording the frequency-following response (FFR), a scalp-recorded electrophysiological response that reflects synchronous activity in subcortical neurons. Marmel et al.^43^ measured the FFR synchronisation strength in response to a 606-Hz pure tone for participants with a wide range of ages and hearing losses. They also measured DLFs for a 606-Hz tone, using a three-alternative forced-choice task (pick the odd one out). They found a small but significant correlation between FFR synchronisation strength and DLFs even when the effects of both absolute thresholds and age were partialled out. These results support the idea that DLFs at low frequencies depend on the use of temporal cues and that individual differences in DLFs partly reflect differences in the precision of phase locking.

## Conclusions

The relative role of place and temporal mechanisms in auditory frequency discrimination was assessed for a centre frequency of 2 kHz. It was hypothesized that: (1) FMDLs depend partly on a temporal mechanism and partly on a place mechanism for an FM rate of 2 Hz, while FMDLs depend primarily on a place mechanism for an FM rate of 20 Hz; (2) DLFs depend primarily on a temporal mechanism; (3) performance of the TFS1 task depends primarily on a temporal mechanism. Based on these hypotheses, four predictions were made: (P1) FM2 and FM20 values should be moderately correlated; (P2) FM2 values should be moderately correlated with DLF and TFS values; (P3) FM20 values should not be correlated with DLF or TFS values; (P4) DLF and TFS values should be highly correlated.

Based on individual differences across 63 young normal-hearing participants, P1, P3 and P4 were supported while P2 was weakly supported. It is suggested that the weak support for P2 was related to the low SL of the stimuli (30 dB), which promoted the use of place cues for FM detection even for the 2-Hz FM rate. A principal components analysis showed that two components accounted for 86% of the variance in the data. PC1 can be interpreted as related to the use of temporal cues, while PC2 can be interpreted as reflecting the use of place cues. Overall, the results support the idea that frequency discrimination at 2 kHz depends partly on the use of temporal cues for FM detection at a 2-Hz rate, mainly on the use of place cues for FM detection at a 20-Hz rate, and mainly on the use of temporal cues for DLFs and TFS. The relative importance of temporal and place cues for higher centre frequencies needs further exploration.

## Data Availability

The data for the current study are available from the corresponding author on reasonable request.
